# 3-Methyl-4-(3-methyl­phen­yl)-5-(2-pyridyl)-4*H*-1,2,4-triazole

**DOI:** 10.1107/S1600536809015712

**Published:** 2009-04-30

**Authors:** Da-Jing Xie, Wei Lu, Zuo-Xiang Wang, Dun-Ru Zhu

**Affiliations:** a College of Chemistry and Chemical Engineering, State Key Laboratory of Materials-Oriented Chemical Engineering, Nanjing University of Technology, Nanjing 210009, People’s Republic of China; b Ordered Matter Science Research Center, Southeast University, Nanjing 210096, People’s Republic of China

## Abstract

In the mol­ecule of the title compound, C_15_H_14_N_4_, the triazole ring is oriented at dihedral angles of 30.8 (2) and 67.4 (2)° with respect to the pyridine and benzene rings, respectively. The crystal structure is stabilized by C—H⋯N hydrogen-bonding inter­actions, forming chains of mol­ecules along [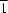
01].

## Related literature

For general background to the chemistry of 1,2,4-triazole derivatives, see: Haasnoot (2000 ▶); Klingele *et al.* (2005 ▶); Moliner *et al.* (2001 ▶). For the applications of iron(II)–triazole complexes in electronics, see: Kröber *et al.* (1993 ▶); Kahn & Martinez (1998 ▶); Zhu *et al.* (2002 ▶). For the synthesis of the title compound, see: Grimmel *et al.* (1946 ▶); Klingsberg *et al.* (1958 ▶). For the synthesis and structures of related triazole ligands and complexes, see: Wang *et al.* (2005 ▶); Liu *et al.* (2005 ▶); Zhu *et al.* (2000 ▶, 2004 ▶, 2005 ▶); Zhang *et al.* (2004 ▶, 2005 ▶); Schneider *et al.* (2007 ▶); Wu *et al.* (2007 ▶); Matouzenko *et al.* (2004 ▶); Nakano *et al.* (2004 ▶); Qi *et al.* (2008 ▶). For bond-length data, see: Allen *et al.* (1987 ▶).
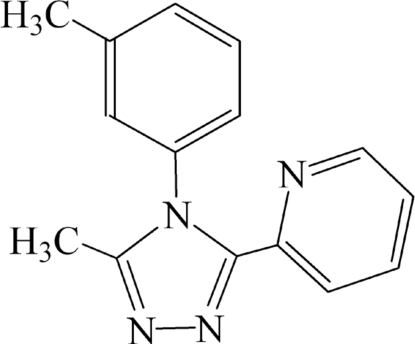


## Experimental

### 

#### Crystal data

C_15_H_14_N_4_
*M* *_r_* = 250.30
Monoclinic, 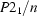

*a* = 9.568 (1) Å
*b* = 10.519 (2) Å
*c* = 13.555 (2) Å
β = 96.64 (3)°
*V* = 1355.1 (4) Å^3^
*Z* = 4
Mo *K*α radiation
μ = 0.08 mm^−1^
*T* = 293 K
0.50 × 0.50 × 0.25 mm

#### Data collection

Bruker APEXII CCD diffractometer
Absorption correction: multi-scan (*SADABS*; Bruker, 2005 ▶) *T*_min_ = 0.963, *T*_max_ = 0.981
10962 measured reflections
2386 independent reflections
1937 reflections with *I* > 2σ(*I*)
*R*_int_ = 0.051

#### Refinement

*R*[*F* ^2^ > 2σ(*F* ^2^)] = 0.057
*wR*(*F* ^2^) = 0.163
*S* = 1.30
2386 reflections
173 parameters
H-atom parameters constrained
Δρ_max_ = 0.15 e Å^−3^
Δρ_min_ = −0.14 e Å^−3^

### 

Data collection: *APEX2* (Bruker, 2005 ▶); cell refinement: *SAINT* (Bruker, 2005 ▶); data reduction: *SAINT*; program(s) used to solve structure: *SHELXS97* (Sheldrick, 2008 ▶); program(s) used to refine structure: *SHELXL97* (Sheldrick, 2008 ▶); molecular graphics: *SHELXTL* (Sheldrick, 2008 ▶); software used to prepare material for publication: *SHELXTL*.

## Supplementary Material

Crystal structure: contains datablocks I, global. DOI: 10.1107/S1600536809015712/rz2308sup1.cif

Structure factors: contains datablocks I. DOI: 10.1107/S1600536809015712/rz2308Isup2.hkl

Additional supplementary materials:  crystallographic information; 3D view; checkCIF report

## Figures and Tables

**Table 1:** Hydrogen-bond geometry (Å, °)

*D*—H⋯*A*	*D*—H	H⋯*A*	*D*⋯*A*	*D*—H⋯*A*
C1—H1*B*⋯N1^i^	0.93	2.56	3.377 (3)	147

Symmetry code: (i) 
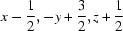
.

**Fig. 1.**
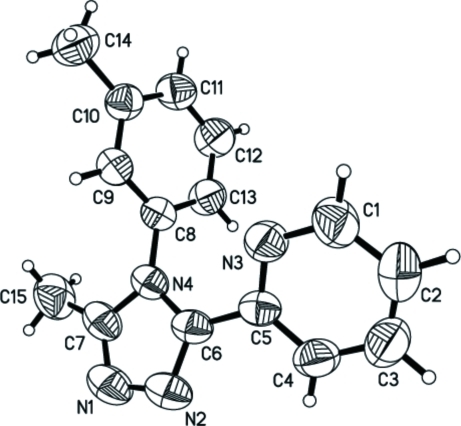
The molecular structure of the title compound with the atomic labelling. Displacement ellipsoids are shown at the 30% probability level

## supplementary crystallographic information

### Comment

In recent years, 1,2,4-triazole derivatives have attracted much attention (Haasnoot 2000; Klingele *et al.*, 2005; Moliner *et al.*, 2001), mainly because of the fact that these molecules can act as flexible bridging ligands and spacers between transition metal ions. For instance, some iron(II) triazole complexes have spin-crossover properties which can be used in molecular electronics (Kröber *et al.*, 1993), as information storage (Kahn & Martinez, 1998) and switching materials (Zhu *et al.*, 2002). Recently, some substituted 1,2,4-triazoles (Wang *et al.*, 2005; Liu *et al.*, 2005; Zhu *et al.*, 2000; Zhang *et al.*, 2004; Zhang *et al.*, 2005) and their metal complexes (Schneider *et al.*, 2007; Wu *et al.*, 2007; Zhu *et al.*, 2004; Matouzenko *et al.*, 2004; Zhu *et al.*, 2005; Nakano *et al.*, 2004; Qi *et al.*, 2008;) have been prepared by us and other groups. We report herein the crystal structure of the title compound, in order to elucidate its molecular conformation.

In the molecule of the title compound (Fig. 1), the bond lengths (Allen *et al.*, 1987) and angles are within normal ranges. The dihedral angle formed by the 1,2,4-triazole ring with the pyridyl and methylphenyl ring is 30.8 (2)° and 67.4 (2)°, respectively. The N3—C5—C6—N4 torsion angle including the N4 atom of the 1,2,4-triazole ring and the N3 atom of the pyridyl ring is 32.0 (3)°. In the crystal packing, chains of molecules running parallel to the [10 1] direction are formed through intermolecular C—H···N hydrogen bonds (Table 1).

### Experimental

The title compoud was synthesized by the reaction of 3,3'-dimethylphenylphosphazoanilide and *N*'-acetyl- N-(2-pyridoyl)hydrazine in *o*-dichlorobenzene at 463–473 K according to the literature method (Grimmel,*et al.* 1946; Klingsberg, *et al.* 1958). Single crystals suitable for X-ray analysis were obtained by recrystallization from an aqeous ethanol solution at room temperature (yield 60%).

### Refinement

All H atoms were located in a difference Fourier map and allowed to ride on their parent atoms, with C—H = 0.93-0.96Å and with *U*_iso_(H) = 1.2*U*_eq_(C) or 1.5*U*_eq_(C) for methyl H atoms.

### Crystal data

**Table:**

C_15_H_14_N_4_	*F*(000) = 528
*M**_r_* = 250.30	*D*_x_ = 1.227 Mg m^−^^3^
Monoclinic, *P*2_1_/*n*	Mo *K*α radiation, λ = 0.71073 Å
Hall symbol: -P 2yn	Cell parameters from 2955 reflections
*a* = 9.568 (1) Å	θ = 2.8–27.5°
*b* = 10.519 (2) Å	µ = 0.08 mm^−^^1^
*c* = 13.555 (2) Å	*T* = 293 K
β = 96.64 (3)°	Prism, colorless
*V* = 1355.1 (4) Å^3^	0.50 × 0.50 × 0.25 mm
*Z* = 4	

### Data collection

**Table:**

Bruker APEXII CCD diffractometer	2386 independent reflections
Radiation source: fine-focus sealed tube	1937 reflections with *I* > 2σ(*I*)
graphite	*R*_int_ = 0.051
ω scans	θ_max_ = 25.0°, θ_min_ = 3.0°
Absorption correction: multi-scan (*SADABS*; Bruker, 2005)	*h* = −11→11
*T*_min_ = 0.963, *T*_max_ = 0.981	*k* = −12→12
10962 measured reflections	*l* = −16→16

### Refinement

**Table:**

Refinement on *F*^2^	Secondary atom site location: difference Fourier map
Least-squares matrix: full	Hydrogen site location: inferred from neighbouring sites
*R*[*F*^2^ > 2σ(*F*^2^)] = 0.057	H-atom parameters constrained
*wR*(*F*^2^) = 0.163	*w* = 1/[σ^2^(*F*_o_^2^) + (0.0801*P*)^2^] where *P* = (*F*_o_^2^ + 2*F*_c_^2^)/3
*S* = 1.30	(Δ/σ)_max_ < 0.001
2386 reflections	Δρ_max_ = 0.15 e Å^−^^3^
173 parameters	Δρ_min_ = −0.14 e Å^−^^3^
0 restraints	Extinction correction: *SHELXL97* (Sheldrick, 2008), Fc^*^=kFc[1+0.001xFc^2^λ^3^/sin(2θ)]^-1/4^
Primary atom site location: structure-invariant direct methods	Extinction coefficient: 0.032 (8)

### Special details

**Table:**

Geometry. All e.s.d.'s (except the e.s.d. in the dihedral angle between two l.s. planes) are estimated using the full covariance matrix. The cell e.s.d.'s are taken into account individually in the estimation of e.s.d.'s in distances, angles and torsion angles; correlations between e.s.d.'s in cell parameters are only used when they are defined by crystal symmetry. An approximate (isotropic) treatment of cell e.s.d.'s is used for estimating e.s.d.'s involving l.s. planes.
Refinement. Refinement of *F*^2^ against ALL reflections. The weighted *R*-factor *wR* and goodness of fit *S* are based on *F*^2^, conventional *R*-factors *R* are based on *F*, with *F* set to zero for negative *F*^2^. The threshold expression of *F*^2^ > σ(*F*^2^) is used only for calculating *R*-factors(gt) *etc*. and is not relevant to the choice of reflections for refinement. *R*-factors based on *F*^2^ are statistically about twice as large as those based on *F*, and *R*- factors based on ALL data will be even larger.

### Fractional atomic coordinates and isotropic or equivalent isotropic displacement parameters (Å^2^)

**Table:**

	*x*	*y*	*z*	*U*_iso_*/*U*_eq_	
N1	0.1372 (2)	0.8645 (2)	0.47665 (12)	0.0792 (6)	
N2	0.05623 (19)	0.76334 (17)	0.50563 (11)	0.0725 (5)	
N3	−0.09930 (17)	0.73930 (15)	0.73296 (12)	0.0658 (5)	
N4	0.11155 (15)	0.88829 (15)	0.63546 (10)	0.0561 (4)	
C1	−0.1707 (2)	0.6566 (2)	0.78336 (17)	0.0793 (7)	
H1B	−0.2143	0.6876	0.8363	0.095*	
C2	−0.1837 (2)	0.5290 (2)	0.76202 (19)	0.0819 (7)	
H2B	−0.2362	0.4761	0.7985	0.098*	
C3	−0.1172 (3)	0.4818 (2)	0.68567 (18)	0.0845 (7)	
H3B	−0.1237	0.3959	0.6694	0.101*	
C4	−0.0409 (2)	0.5632 (2)	0.63355 (15)	0.0731 (6)	
H4A	0.0058	0.5328	0.5819	0.088*	
C5	−0.03405 (18)	0.69079 (18)	0.65844 (12)	0.0537 (5)	
C6	0.04303 (19)	0.77911 (17)	0.60035 (13)	0.0559 (5)	
C7	0.1694 (2)	0.9368 (2)	0.55513 (15)	0.0680 (6)	
C8	0.12918 (18)	0.93671 (17)	0.73586 (13)	0.0527 (5)	
C9	0.06763 (19)	1.05012 (18)	0.75777 (13)	0.0571 (5)	
H9A	0.0163	1.0962	0.7074	0.069*	
C10	0.0816 (2)	1.09668 (18)	0.85510 (14)	0.0608 (5)	
C11	0.1585 (2)	1.0247 (2)	0.92840 (15)	0.0719 (6)	
H11A	0.1680	1.0531	0.9938	0.086*	
C12	0.2210 (2)	0.9120 (2)	0.90560 (16)	0.0793 (7)	
H12A	0.2728	0.8657	0.9557	0.095*	
C13	0.2075 (2)	0.86690 (19)	0.80930 (14)	0.0673 (6)	
H13A	0.2501	0.7911	0.7940	0.081*	
C14	0.0136 (3)	1.2195 (2)	0.88081 (18)	0.0886 (7)	
H14A	0.0346	1.2357	0.9507	0.133*	
H14B	−0.0864	1.2134	0.8642	0.133*	
H14C	0.0493	1.2879	0.8440	0.133*	
C15	0.2596 (3)	1.0526 (2)	0.55930 (19)	0.0941 (8)	
H15A	0.2877	1.0689	0.4948	0.141*	
H15B	0.3416	1.0395	0.6061	0.141*	
H15C	0.2075	1.1240	0.5797	0.141*	

### Atomic displacement parameters (Å^2^)

**Table:**

	*U*^11^	*U*^22^	*U*^33^	*U*^12^	*U*^13^	*U*^23^
N1	0.0814 (13)	0.1051 (15)	0.0546 (11)	0.0076 (11)	0.0229 (9)	0.0042 (9)
N2	0.0765 (11)	0.0929 (13)	0.0501 (10)	0.0047 (9)	0.0158 (8)	−0.0068 (8)
N3	0.0660 (10)	0.0654 (10)	0.0701 (11)	−0.0030 (8)	0.0257 (8)	−0.0060 (8)
N4	0.0555 (9)	0.0664 (10)	0.0474 (9)	0.0024 (7)	0.0109 (7)	0.0019 (7)
C1	0.0745 (14)	0.0799 (16)	0.0892 (16)	−0.0026 (11)	0.0344 (12)	0.0051 (11)
C2	0.0766 (15)	0.0713 (15)	0.0959 (18)	−0.0122 (11)	0.0024 (12)	0.0183 (12)
C3	0.1042 (19)	0.0598 (13)	0.0850 (17)	−0.0060 (13)	−0.0078 (14)	−0.0009 (12)
C4	0.0935 (16)	0.0646 (13)	0.0602 (13)	0.0125 (11)	0.0040 (11)	−0.0104 (10)
C5	0.0512 (10)	0.0624 (11)	0.0466 (10)	0.0045 (8)	0.0022 (7)	−0.0059 (8)
C6	0.0544 (10)	0.0672 (12)	0.0466 (10)	0.0076 (9)	0.0084 (8)	−0.0056 (8)
C7	0.0651 (12)	0.0856 (14)	0.0556 (12)	0.0039 (10)	0.0173 (9)	0.0108 (10)
C8	0.0508 (10)	0.0598 (11)	0.0477 (10)	−0.0027 (8)	0.0060 (8)	−0.0003 (8)
C9	0.0549 (11)	0.0626 (11)	0.0541 (11)	0.0017 (9)	0.0075 (8)	0.0057 (8)
C10	0.0599 (11)	0.0611 (12)	0.0616 (12)	−0.0053 (9)	0.0067 (9)	−0.0054 (9)
C11	0.0791 (14)	0.0800 (14)	0.0534 (12)	−0.0072 (11)	−0.0057 (10)	−0.0088 (10)
C12	0.0842 (15)	0.0839 (15)	0.0633 (14)	0.0113 (12)	−0.0186 (11)	−0.0015 (11)
C13	0.0692 (13)	0.0658 (12)	0.0637 (13)	0.0106 (10)	−0.0053 (10)	−0.0023 (9)
C14	0.1013 (18)	0.0802 (16)	0.0844 (16)	0.0122 (13)	0.0109 (13)	−0.0157 (12)
C15	0.0968 (18)	0.0993 (18)	0.0908 (17)	−0.0152 (14)	0.0309 (14)	0.0164 (13)

### Geometric parameters (Å, °)

**Table:**

N1—C7	1.315 (3)	C8—C9	1.378 (3)
N1—N2	1.399 (3)	C8—C13	1.385 (3)
N2—C6	1.315 (3)	C9—C10	1.399 (3)
N3—C1	1.341 (3)	C9—H9A	0.9300
N3—C5	1.348 (2)	C10—C11	1.390 (3)
N4—C7	1.375 (3)	C10—C14	1.506 (3)
N4—C6	1.380 (3)	C11—C12	1.379 (3)
N4—C8	1.445 (3)	C11—H11A	0.9300
C1—C2	1.376 (4)	C12—C13	1.381 (3)
C1—H1B	0.9300	C12—H12A	0.9300
C2—C3	1.369 (3)	C13—H13A	0.9300
C2—H2B	0.9300	C14—H14A	0.9600
C3—C4	1.373 (3)	C14—H14B	0.9600
C3—H3B	0.9300	C14—H14C	0.9600
C4—C5	1.384 (3)	C15—H15A	0.9600
C4—H4A	0.9300	C15—H15B	0.9600
C5—C6	1.470 (3)	C15—H15C	0.9600
C7—C15	1.490 (4)		
			
C7—N1—N2	107.35 (17)	C13—C8—N4	118.98 (18)
C6—N2—N1	107.28 (17)	C8—C9—C10	120.65 (17)
C1—N3—C5	116.33 (19)	C8—C9—H9A	119.7
C7—N4—C6	104.75 (17)	C10—C9—H9A	119.7
C7—N4—C8	126.95 (19)	C11—C10—C9	117.9 (2)
C6—N4—C8	128.13 (15)	C11—C10—C14	120.6 (2)
N3—C1—C2	124.4 (2)	C9—C10—C14	121.47 (19)
N3—C1—H1B	117.8	C12—C11—C10	121.0 (2)
C2—C1—H1B	117.8	C12—C11—H11A	119.5
C3—C2—C1	118.3 (2)	C10—C11—H11A	119.5
C3—C2—H2B	120.9	C11—C12—C13	120.76 (19)
C1—C2—H2B	120.9	C11—C12—H12A	119.6
C2—C3—C4	119.0 (2)	C13—C12—H12A	119.6
C2—C3—H3B	120.5	C12—C13—C8	118.8 (2)
C4—C3—H3B	120.5	C12—C13—H13A	120.6
C3—C4—C5	119.5 (2)	C8—C13—H13A	120.6
C3—C4—H4A	120.2	C10—C14—H14A	109.5
C5—C4—H4A	120.2	C10—C14—H14B	109.5
N3—C5—C4	122.48 (18)	H14A—C14—H14B	109.5
N3—C5—C6	117.83 (18)	C10—C14—H14C	109.5
C4—C5—C6	119.66 (18)	H14A—C14—H14C	109.5
N2—C6—N4	110.26 (17)	H14B—C14—H14C	109.5
N2—C6—C5	123.65 (18)	C7—C15—H15A	109.5
N4—C6—C5	126.10 (17)	C7—C15—H15B	109.5
N1—C7—N4	110.4 (2)	H15A—C15—H15B	109.5
N1—C7—C15	125.7 (2)	C7—C15—H15C	109.5
N4—C7—C15	123.9 (2)	H15A—C15—H15C	109.5
C9—C8—C13	120.88 (18)	H15B—C15—H15C	109.5
C9—C8—N4	120.14 (16)		

### Hydrogen-bond geometry (Å, °)

**Table:**

*D*—H···*A*	*D*—H	H···*A*	*D*···*A*	*D*—H···*A*
C1—H1B···N1^i^	0.93	2.56	3.377 (3)	147

Symmetry codes: (i) *x*−1/2, −*y*+3/2, *z*+1/2.
